# Epigenetic modifications potentially controlling the allelic expression of imprinted genes in sunflower endosperm

**DOI:** 10.1186/s12870-021-03344-4

**Published:** 2021-12-04

**Authors:** Zhichao Zhang, Shuai Yu, Jing Li, Yanbin Zhu, Siqi Jiang, Haoran Xia, Yue Zhou, Daqiu Sun, Meiling Liu, Cong Li, Yanshu Zhu, Yanye Ruan, Xiaomei Dong

**Affiliations:** 1 College of Bioscience and Biotechnology, Shenyang Agricultural University, Shenyang, 110866 Liaoning China; 2 Shenyang City Key Laboratory of Maize Genomic Selection Breeding, Shenyang, 110866 Liaoning China; 3 School of Traditional Chinese Materia Medica, Shenyang Pharmaceutical University, Shenyang, China; 4 State Key Laboratory of Maize Bio-Breeding, Shenyang, China; 5 State Key Laboratory of the Northeast Crop Genetics and Breeding, Shenyang, China

**Keywords:** Genomic imprinting, DNA methylation, Endosperm, Sunflower

## Abstract

**Background:**

Genomic imprinting is an epigenetic phenomenon mainly occurs in endosperm of flowering plants. Genome-wide identification of imprinted genes have been completed in several dicot Cruciferous plant and monocot crops.

**Results:**

Here, we analyzed global patterns of allelic gene expression in developing endosperm of sunflower which belongs to the composite family. Totally, 691 imprinted loci candidates were identified in 12 day-after-pollination sunflower endosperm including 79 maternally expressed genes (MEG) and 596 paternally expressed genes (PEG), 6 maternally expressed noncoding RNAs (MNC) and 10 paternally expressed noncoding RNAs (PNC). And a clear clustering of imprinted genes throughout the rapeseed genome was identified. Generally, imprinting in sunflower is conserved within a species, but intraspecific variation also was detected. Limited loci in sunflower are imprinted in other several different species. The DNA methylation pattern around imprinted genes were investigated in embryo and endosperm tissues. In CG context, the imprinted genes were significantly associated with differential methylated regions exhibiting hypomethylation in endosperm and hypermethylation in embryo, which indicated that the maternal demethylation in CG context potentially induce the genomic imprinting in endosperm.

**Conclusion:**

Our study would be helpful for understanding of genomic imprinting in plants and provide potential basis for further research in imprinting in sunflower.

**Supplementary Information:**

The online version contains supplementary material available at 10.1186/s12870-021-03344-4.

## Background

Genomic imprinting is an epigenetic phenomenon in flowering plants and mammals that relies on chromatin modifications established in the male and female gametogenesis, resulting in the differential expression of the maternally- or the paternally-inherited allele [1, 2]. Genomic imprinting has been observed in many species, from mammals to flowering plants [3–10]. In plant, most genomic imprinting occurs in endosperm which plays an important role in coordinating embryo and seed growth [11–15]. Imprinted genes have been shown to play key roles in many biological progresses of seed development, postzygotic interploidy hybridization and germination processes [13–16]. Hence, identification of imprinted genes and disclosing these gene’s function play important roles in understanding seed development in plant.

Deep sequencing of RNA-seq libraries of reciprocal crosses from endosperm has been used successfully to identify imprinted genes [3–8, 17–21]. However, although assembled and annotated genomes are available for several hundred flowering plants, genome-wide identification of imprinted genes were fulfilled in only limited plants. Especially for dicot plant, the imprinted genes were intensively identified in Cruciferous family [4, 6, 17, 21]. The conservation of genomic imprinting was shown to be relatively low in plant [21–23]. Hence, Genome-wide screening and analysis of imprinted genes is to be studied in other species.

In both plants and mammals, DNA methylation is one of the primary modifications associated with genomic imprinting [24, 25]. In *A. thaliana*, DNA methylation close to several maternally expressed genes (MEGs), including FWA, FIS2, and MPC, was shown to be important for their maternally preferred expression because all these genes exhibited biallelic expression in endosperm fertilized with *met1* pollen [26–28]. Genome-wide analysis of allele-specific DNA methylation had been reported in several plant species [8, 29, 30], and parent-of-origin dependent differentially methylated regions were found to be significantly associated with imprinted genes [31]. However, the regulation mechanism of DNA methylation for genomic imprinting is different. In *A. thaliana*, the silent maternal alleles of paternally expressed genes (PEGs) were CG hypermethylated in the gene body [17]. Whereas, the maternal alleles of many *A. lyrata* PEGs were hypermethylated in the CHG context [19]. These findings suggest that imprinting mechanisms may be flexible on relatively short evolutionary time scales.

The domesticated sunflower (*Helianthus annuus* L.), belongs to family Asteraceae, which is one of the largest in the world with members across all continents [32]. *H. annuus* L. is a large annual forb of genus *Helianthus* that is grown as a crop for its edible oil and fruits. And sunflower seeds are among the top five most abundant oilseeds that are grown worldwide [33]. The endosperm tissue can be easily separated from embryo and other maternal tissues to avoid contamination, due to a single large head and large seeds in modern domesticated sunflowers. In this study, we analyzed large-scale sequencing data of sunflower endosperm transcriptomes to comprehensively identify imprinted transcripts (including protein-coding genes and noncoding RNAs), and found that imprinted loci were usually clustered in the genome. The association between DNA methylation and the imprinted genes also was analyzed to explore the mechanism of epigenetic regulation for genomic imprinting in sunflowers. Together, our findings will enrich the knowledge of genomic imprinting in flowering plants and will be helpful for further research on how genomic imprinting regulates endosperm development.

## Results

### Genome-wide screening of putative imprinted genes in sunflower endosperm

To assess the allelic expression patterns of genes in sunflower endosperm, deep sequencing of RNA isolated from endosperm tissue 12 days after pollination (DAP) of reciprocal hybrid pairs was performed to identify imprinted genes. To increase the efficiency of hybridization and allow rigorous control of the timing of fertilization to avoid false hybridization events, we used three-line hybrids to construct reciprocal crosses. Cytoplasmic male sterile (CMS) lines have the same nuclear genome as the corresponding maintainer lines, but different cytoplasmic genomes. Here, we used the CMS line 138A with its maintainer line 138B and the CMS line 398A with its maintainer line 398B to make reciprocal crosses, which we called 138A × 398B (denoted as SY1) and 398A × 138B (YS1), and the CMS line 723A with its maintainer line 723B and the CMS line 6A with its corresponding maintainer line 6B to make reciprocal crosses, which we called 723A × 6B (SY2) and 6A × 723B (YS2).

**Fig. 1:**
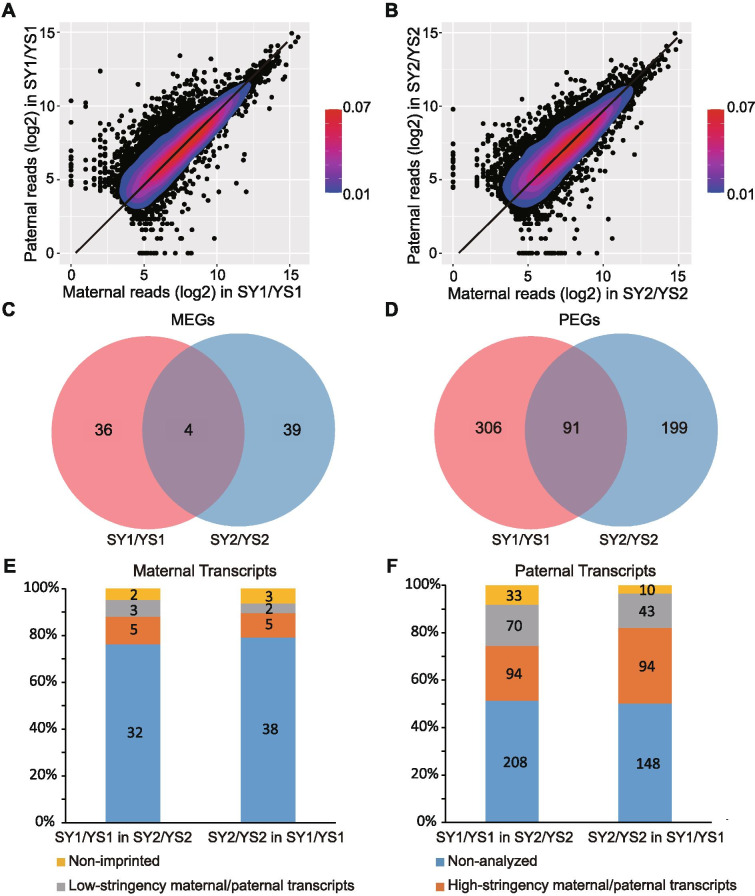
Identification of imprinted genes in sunflower endosperm at 12 DAP. **A** and **B** Parental expression ratios plot for each reciprocal cross in SY1/YS1 (**A**) and SY2/YS2 (**B**). The expression levels of paternal (y-axis) and maternal (x-axis) alleles are represented by the log_2_ read counts of the paternally- and maternally-derived reads in the reciprocal crosses, respectively. The color scale in blue (low) and red (high) represents the relative density of the genes. The solid diagonal line represents the expected 2 m:1p ratio. **C** and **D** Venn diagram analysis of imprinted genes. The number of imprinted genes identified in two crosses are shown in the red (SY1/YS1) and blue (SY2/YS2) circles, respectively. **E** and **F** Comparison of imprinted genes in two crosses of sunflower. Non-imprinted: genes not showing significant deviation from 2:1 ratio of maternal allele to paternal allele in each reciprocal hybrid (Chi-square (2:1, *q* > 0.05)). Non-analyzed: genes without sufficient read counts. Low-stringency maternal transcripts (MEG and MNC)/paternal transcripts (PEG and PNC): transcripts showing significant deviation from 2:1 ratio of maternal allele to paternal allele in each reciprocal hybrid (Chi-square (2:1, *q* < 0.05)). High-stringency MEGs/PEGs: genes in which favorable alleles were at least five times more than those of non-favorable alleles in both directions of a reciprocal cross (*q* < 0.05 (χ2 test))

By analyzing the data obtained by the deep high-throughput re-sequencing of endosperm libraries constructed from four parents (Additional file 1: Table S1), we identified a total of 1,022,533 and 839,168 SNPs between the 398A and 138A lines, and the 723A and 6A lines, respectively (see Materials and methods). First, the RNA-seq reads of the 12 DAP endosperm from two parent lines (398A and 138A) were mapped to genome (Additional file 1: Table S1). The reads ratio between the 138A and 398A alleles at each SNP site were calculated to test the accuracy of the identified SNPs between the 398A and 138A lines. We found that 99.1 and 99.3% of the reads were from 138A and 398A alleles, respectively (Additional file 7: Fig. S1), which indicate that the SNPs identified between these parents were high-quality. Then, the RNA-seq reads of the 12 DAP endosperm from the hybrids of reciprocal crosses (SY1/YS1 and SY2/YS2) were mapped to genome for identification of imprinted genes (Additional file 1: Table S1), and a total of 17,420 and 17,954 expressed genes (FPKM ≥1) were identified from SY1/YS1 and SY2/YS2, respectively. We found that most of the allelically analyzed genes showed bi-allelic expression in the hybrid endosperm (Fig. 1a, b). Genes with parental allele bias (q < 0.05 (χ2 test)) in reciprocal hybrids were classified as low-stringency imprinted genes. We identified 858 (266 MEGs and 592 PEGs) and 770 (305 MEGs and 465 PEGs) imprinted genes in SY1/YS1 and SY2/YS2, respectively. Under the criteria, we further defined high-stringency MEGs and PEGs in which favorable alleles were at least five times more than those of non-favorable alleles in both directions of a reciprocal cross (q < 0.05 (χ2 test)). Using these criteria, we identified 40 and 397 high-stringency MEGs and PEGs, respectively, in the SY1/YS1 endosperm, and 43 and 290 high-stringency MEGs and PEGs, respectively, in the SY2/YS2 endosperm (Additional file 2: Table S2). Furthermore, according to the annotation of long noncoding RNAs in the sunflower genome [32], we identified two maternally expressed noncoding RNAs (MNCs) and eight paternally expressed noncoding RNAs (PNCs) in the SY1/YS1 endosperm, and five MNCs and five PNCs in the SY2/YS2 endosperm (Additional file 2: Table S2). The GO biological process annotations of the imprinted genes were assessed. Gene ontology analysis showed that imprinted genes are enriched in binding, carbohydrate binding, protein binding and drug binding according to their molecular function and macromolecule localization, signal transduction, and signaling according to their biological processes (Additional file 8: Fig. S2).

Maternal contamination is a known issue with endosperm RNA-seq data for identifying imprinted genes [34]. For 12DAP sunflower, the seed were enough large with about 9 mm length. The embryo and endosperm with about 5 mm length were easily separated from seed coat. As a result, only 12% of genes exhibited parental allele bias (*P*-value cutoff of 0.05 (χ2 test)) in endosperm. And the proportion for number of MEGs were significantly lower compared with number of PEG. Next, we identified homologs for four seed coat-specific genes in Arabidopsis including PER36, MUM4, COBAL-like2 and COBAL-like 3[35, 36] Indeed, the sunflower homologs for four seed coat-specific genes in Arabidopsis were not expressed in endosperm (with FPKM < 0.5). Hence, The results indicated that the endosperm were sampled without contamination.

### Comparison of imprinted genes identified in two sunflower crosses

Some of the examples of imprinted loci exhibited imprinting of alleles from some genotypes but not others. We assessed allelic imprinting variation in the sunflowers and detected high-stringency imprinted loci that overlapped in the two crosses (SY1/YS1 and SY2/YS2) as visualized in the Venn diagram (Fig. 1c, d). Although 4 MEGs and 91 PEGs were found to overlap in the two crosses, most of the imprinted genes identified in one cross tended to be imprinted in other reciprocal crosses (Fig. 1e, f). Imprinted genes found in only one set of reciprocal crosses usually lacked informative SNPs or had insufficient reads to identify if they were imprinted in other crosses (Fig. 1c, d). For example, among the 405 paternal transcripts (including 397 PEGs and 8 PNCs) identified in the SY1/YS1 endosperm, 164 were PEGs/PNCs, 33 were bi-allelically expressed, and 208 (51.4%) had no polymorphisms or were not expressed in SY2/YS2 endosperm. Overall, we found that many genes (81.9%) that exhibited imprinting in one cross were also imprinted in the other crosses. However, some genes were imprinted in one cross displayed allelic variation for imprinting in another cross.

### Imprinted genes tend to be clustered in the genome

**Fig. 2:**
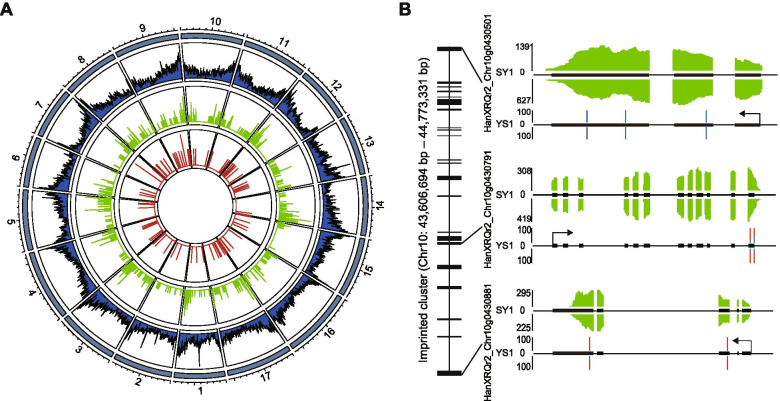
Characteristics of the sunflower imprinted transcripts. **A** Distribution of the sunflower all genes (outer, blue), PEGs (middle, green) and MEGs (inner, red) along each chromosome. **B** One 1.17 Mb region (Chr10: 43,606,694 bp – 44,773,331 bp) were shown. The region including one MEG, two PEGs, five non-imprinted genes and 31 non-analyzed genes. The black rectangle represent the location of these 39 genes within cluster. The expression of one MEG (HanXRQr2_Chr11g0506811) and two PEGs (HanXRQr2_Chr11g0506781 and HanXRQr2_Chr11g0507231) were shown. The expression level of transcribed regions is shown in green for SY1 and YS1; The percentages of allelic reads of three imprinted genes for specific SNP sites are shown, with red lines for the paternal allele and blue lines for the maternal allele; Black rectangle, exon; black line, intron

Imprinted genes in human and mouse tend to cluster in their genomes [37]. The relatively large number of imprinted transcripts identified in this study allowed us to test whether the imprinted genes in sunflower also were clustered in the sunflower genome. We identified a total of 691 imprinted transcripts (79 MEGs, 6 MNCs, 596 PEGs, 10 PNCs) in sunflower endosperm from two reciprocal hybrid pairs (Fig. 2a), and the average distance between them was 4.14 Mb (Additional file 9: Fig. S3). Here we showed an approximately 1.17 Mb region including one MEG and two PEGs (Fig. 2b), SNPs in the MEGs exhibited significant maternal bias and SNPs in the PEGs exhibited significant paternal bias.

We scanned the genome for candidate clusters containing at least two imprinted transcripts within a region of 1 Mb and all allelically analyzed genes located within clusters were imprinted genes, a standard that is similar to that used in mammals [10]. A total of 195 imprinted genes (including 8 MEGs and 187 PEGs) fell into 85 clusters (Additional file 3: Table S3). The 76.5, 17.6 and 5.9% of clusters contained two, three and four imprinted genes, respectively. Except for three imprinted cluster including both MEG and PEG, most of imprinted genes in one cluster showed same parental preference. The average distance of imprinted genes in cluster were 25.6 kb. Some imprinted genes were very close in genome. For example, PEGs HanXRQr2_Chr11g0470191 annotated as “transcription factor C2H2r” and HanXRQr2_Chr11g0470201 annotated as “serine/threonine phosphatase” were approximately 1 kb apart.

### Conservation of imprinted genes

We systematically identified imprinted genes in the endosperm of sunflower, which enabled us to better analyze the conservation of imprinted genes among sunflower and other plants. We compared the imprinted genes in sunflower with those in dicotyledons (dicots) such as *A. thaliana*, castor bean, and *C. rubella*, and monocotyledons (monocots) such as maize, rice, and sorghum. We found that 64.7–94.3% of imprinted genes in the six species had homologs in sunflower (Additional file 4: Table S4). Among the homologs, 54.1–91.9% of the imprinted genes were allelically analyzed in sunflower. Of the *A. thaliana* MEGs and PEGs for which there was sufficient sunflower data, 16.2% (31/191) and 27.5% (14/51), respectively, exhibited also maternally and paternally bias expression in sunflower. Among them, 3.7% (7/191) and 25.5% (13/51) were high-stringency MEGs and PEGs in both species. Of the maize MEGs and PEGs for which sufficient data were available in sunflower, 15.4% (6/39) and 23.8% (35/147) exhibited maternally and paternally bias expression in sunflower, with 5.1% (2/39) and 12.2% (18/147) being high-stringency MEGs and PEGs in both species. The results for the other four species, caster bean, *C. rubella*, rice, and sorghum, were also analyzed (Additional file 4: Table S4). These results indicated that a limited number of imprinted loci in sunflower were conserved in the other species. Although sunflower and cruciferous plants are dicots, the conservation of imprinting between sunflower and the cruciferous plants was almost same as that between sunflower and the monocots. Furthermore, the conservation in the PEGs was significantly higher than that in the MEGs, except for cater bean. However, the limited number PEGs in cater bean may have influenced this statistic.

### Expression of imprinted genes among tissues

Most imprinted genes in plants have been found to show specific or preferential expression in endosperm [4, 7, 17, 18]. To explore the expression patterns of the sunflower imprinted genes, we downloaded and analyzed publicly available RNA-Seq data sets of other sunflower tissues, including pistil, stamen, ligule, mature leaf, root, and seed. We found that approximately 62% (49/79) and 39.1% (233/596) of the MEGs and PEG, respectively, were specifically or preferentially expressed in the sunflower endosperm. More MEGs than PEGs were preferentially expressed, implying that most of the sunflower PEGs were not functionally restricted to the endosperm (Additional file 10: Fig. S4). We divided the imprinted genes into subgroups according to their tissue-specificity, and identified 30 constitutive MEGs (con-MEGs), 49 endosperm-specific MEGs (endo-MEGs), 363 constitutive PEGs (con-PEGs), and 233 endosperm-specific PEGs (endo-PEGs). The expression levels of endo-MEGs and endo-PEGs were higher than those of con-MEGs and con-PEGs, respectively. Compared with the expression levels of all the genes in endosperm, the expression levels of most of the imprinted genes were similar, expect for the con-MEGs (Additional file 11: Fig. S5). The result seems to be accorded well with the previous study in maize that con-MEGs may only expressed in a small area of endosperm [38].

### Relationship of imprinted genes and DNA methylation

**Fig. 3:**
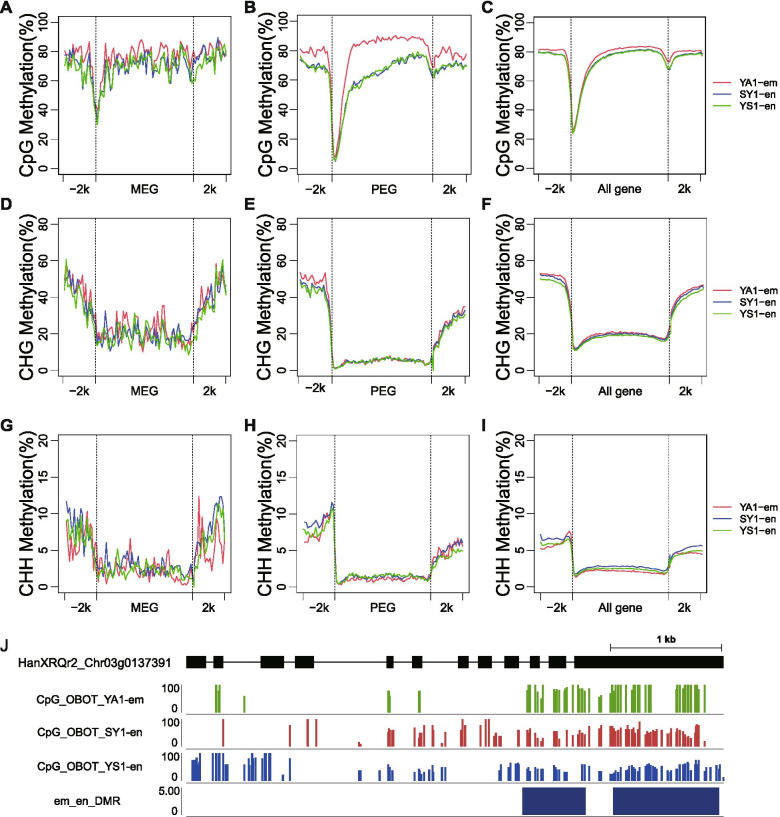
The DNA methylation pattern at imprinted genes. **A**-**C** Average DNA methylation levels of MEGs, PEGs, and all genes for embryo, and endosperm in CpG context throughout the gene body and its 2-kb up- and downstream regions. **D**-**F** Average DNA methylation levels of MEGs, PEGs, and all genes for embryo, and endosperm in CHG context throughout the gene body and its 2-kb up- and downstream regions. **G**-**I** Average DNA methylation levels of MEGs, PEGs, and all genes for embryo, and endosperm in CHH context throughout the gene body and its 2-kb up- and downstream regions. **J** Views of DNA methylation at a PEG (HanXRQr2_Chr03g0137391). The DNA methylation level of embryo (398A), endosperm (SY1 and YS1) at transcribed regions is shown in green, red and blue lines, respectively. Purple rectangle, DMR; Black rectangle, exon; black line, intron

Genomic imprinting is often associated with epigenetic modifications [39, 40]. In this study, we performed bisulfite sequencing of genomic DNA from sunflower endosperm and embryos at 12 DAP to quantify their methylation profiles (Additional file 1: Table S1). The average CG, CHG, and CHH methylation levels were 84.3, 69.5, and 3.4% in embryo, and 84.5, 67.1 and 5.5% in endosperm. The overall methylation level in endosperm was similar to those in embryo (Additional file 12: Fig. S6). The availability of imprinted gene information and whole genome DNA methylome data allowed us to investigate the correlation between DNA methylation and expression of the imprinted genes. In the CG context, the overall DNA methylation levels of PEGs in the upstream, downstream, and gene body regions were slightly lower in endosperm than they were in embryo (Fig. 3a–c), and the DNA methylation levels of MEGs and all genes were similar between endosperm and embryo (Fig. 3a–c). In the CHG and CHH contexts, the overall DNA methylation levels for MEGs, PEGs, and all genes were similar between endosperm and embryo (Fig. 3d–i). Representative examples of the CG methylation for the entire gene body, and upstream and downstream regions are shown for one PEG, HanXRQr2_Chr03g0137391 (Fig. 3j). The DNA methylation levels were lower in the 5′ regions of the gene bodies of the PEGs in embryo compared with the levels in endosperm (Fig. 3j).

Next, we systematically identified differentially methylated regions (DMRs) between tissues in the CG context (Additional file 5: Table S5) and analyzed the relationship between the sunflower imprinted genes and the DMRs. Compared with all genes, the PEGs and MEGs were highly correlated with the occurrence of DMRs that were hypomethylated in endosperm and hypermethylated in embryo. Among 223 PEGs (including their 2-kb up- and downstream regions) that overlapped with the analyzed methylated regions, 56 (27.9%) had DMRs that were hypomethylated in endosperm and hypermethylated in embryo (Additional file 6: Table S6). Similarly, among 10 MEGs (including their 2-kb up- and downstream regions) that overlapped with the analyzed methylated regions, 2 (20%) had DMRs that were hypomethylated in endosperm and hypermethylated in embryo (Additional file 6: Table S6). Overall, the imprinted genes were significantly associated with DMRs that were hypomethylated in endosperm and hypermethylated in embryo (Fisher test; *P*-value < 2.2e− 16), and there was no overlap between imprinted genes and DMRs that were hypomethylated in embryo and hypermethylated in endosperm.

## Discussion

In recent years, studies about genomic imprinting have been intensified to focus on genomic interactions between the maternal and paternal genomes control endosperm development in plant, especially in several important crops [3, 5, 7, 20, 22]. At present, with the exception of Cruciferae species, no studies on genomic imprinting in the endosperm of dicots are currently available [4, 17]. Sunflower is an important oil crop and genome-wide identification of imprinted genes in sunflower endosperm may help to improve the understanding of storage accumulation and provide another model system for seed development.

In this study, we identified 691 imprinted loci by generating reciprocal crosses of different sunflower lines (Additional file 1: Table S1). We found that 28.2% of the identified imprinted genes in sunflower formed 85 distinct clusters in the sunflower genome, which is higher than the proportion of imprinted genes in clusters reported in Arabidopsis and rice. This finding suggests that clustering may play a role in imprinted gene regulation in sunflower. Allele-specific imprinting has been found in plants [22, 41]. Here, we discovered 48 imprinted genes that showed parent-of-origin-dependent expression in certain genotypes but not in others (Additional file 3: Table S3). This finding indicated that imprinting of some important genes may be different in different crosses.

Conservation of imprinting among species is an index related to the importance of the functions of imprinted genes. In mammals, more than 90% of imprinted genes are conserved between mouse and human, whereas, in plants, conservation of imprinted genes was found to be relatively low [42–44]. We identified conserved imprinted genes between any pairs among the monocots (maize, rice, sorghum) and found that 2.0–28.7% of the imprinted genes were conserved. Of the conserved imprinted genes that we identified between any pairs among the dicots (castor bean, *C. rubella*, *A. thaliana*) only 8 were conserved between *A. thaliana* and castor bean, and approximately 20% of the *C. rubella* imprinted genes showed monoallelic expression in *A. thaliana*. We compared the sunflower imprinted genes with those identified in the other six plant species and found that approximately 13.0–26.4% of the sunflower imprinted genes had imprinted homologs in at least one of the six species. This finding is consistent with previous estimates that about 20% of imprinted genes are conserved between species. Unfortunately, the sunflower homologs of the most studied imprinted genes in Arabidopsis, such as *FIS2*, *FIE1*, and *MEA*, could not be confirmed because they lacked SNPs in sunflower. But a sunflower PEG (HanXRQr2_Chr17g0788481) was conserved in rice, maize, *A. thaliana*, and *C. rubella*. The PEG (HanXRQr2_Chr17g0788481) homologs were VIM5 in *A. thaliana*, Carubv10019919mg in *C. rubella*, vim104 in maize, and Os04g22240 in rice. In Arabidopsis, mutants of *VIM5* had defective endosperm [45, 46]. Hence, reverse genetic studies that focus of conserved imprinted genes among grass species may provide insights into their functional roles in seed development.

The regulation of DNA methylation in controlling imprinting may be different in plants. In *A. thaliana*, the maternal alleles of CG demethylation in the gene body were associated with PEGs [17]. In *A. lyrate*, the maternal demethylation occur at body and up-stream of PEGs only in CHG context [19]. In maize, the maternal demethylation of PEGs was discovered in CG and CHG context. In sunflower, our study indicated that the maternal demethylation occurs at body and up−/down-stream of PEGs only in CG context. These studies suggest that the epigenetic modifications potentially controlling the allelic expression of imprinted genes were indeed flexibility in plants.

Recent studies have shown that unsuccessful imprinting leads to endosperm incompatibilities, resulting in failure of interspecific and ploidy hybridization [47, 48]. Wild sunflower species of are valuable resources for genetic variation of cultivated sunflower species. Some wild sunflower species have resistance genes to the main diseases and pests of cultivated sunflower species. Interspecific hybridization allows the resistance genes carried by wild sunflower species to be transferred to cultivated species to obtain resistant materials and to breed disease-resistant varieties. However, interspecific hybridization is a distant hybridization and is often unsuccessful. One of the main reasons for this is embryo abortion because of early endosperm incompatibilities. Hence, knowledge of the imprinted genes and their regulation in cultivated species may help to better understand the underlying mechanisms behind these endosperm-based barriers in the future.

## Conclusion

Here we reported nearly 700 high-stringency imprinted genes in the domesticated sunflower belonging to Asteraceae family, which is one of the largest family in the world. The GO terms analysis of imprinted genes were enriched in specific some functional groups, which indicated that the importance role of these genes in endosperm development. The imprinting conservation were limited between sunflower and other several different species, is conserved within a species, but some well-known imprinted genes in other species also was detected. The association between DNA methylation and the imprinted genes also was analyzed to explore the mechanism of epigenetic regulation for genomic imprinting in sunflowers. These results extend our understanding of gene imprinting in plants and provide potential avenues for further research in imprinted genes.

## Materials and methods

### Plant tissue collection

The CMS line 138A and its maintainer line 138B, the CMS line 398A and its maintainer line 398B, the CMS line 723A and its maintainer line 723B, the CMS line 6A and its corresponding maintainer line 6B, were provided by Shenyang east Asia seed industry, and grown in the filed in Shenyang, Liaoning, China. The 138A and 723 A are domesticated as edible fruit. The 398A and 6A are domesticated for oil. Reciprocal crosses 138A × 398B (denoted as SY1), 398A × 138B (YS1), 723A × 6B (SY2) and 6A × 723B (YS2) were carried out. The embryo and endosperm were collected at 12 days after pollination. Each type of sample was collected from at least three plants. The pericarp and seed coat were peeled rapidly with scalpel. The embryo (white) first was immediately collected on the top using the prepared tweezers. Embryo was washed three times with RNA-free water within 5 min and was quickly transferred into EP tube filled with lipid nitrogen. Then we gently pick out endosperm (pellucidity) and was quickly transferred into EP tube filled with lipid nitrogen.

### DNA and RNA isolation

DNA was extracted using a commercial kit (Super Plant Genomic DNA Kit (Polysacchardes & Polyphenolics-rich)). Total RNA was extracted from the endosperm and embryo of sunflowers using a commercial kit (RNA prep Pure Plant Plus Kit (Polysaccharides & Polyphenolics-rich)). DNA quantity and RNA quantity were measured using a BioDrop (BioDrop μLite+). RNA degradation and contamination was monitored on agarose gels. RNA purity was checked using the spectrophotometer. RNA concentration was measured using Qubit. RNA integrity was assessed using the Agilent 2100. A total amount of 2 μg RNA per sample was used as input material for the RNA sample preparations. Sequencing libraries were generated using VAHTS mRNA-seq v2 Library Prep Kit for Illumina following manufacturer’s recommendations and index codes were added to attribute sequences to each sample. Briefly, mRNA was purified from total RNA using poly-T oligo-attached magnetic beads. Fragmentation was carried out using fragmentation buffer. First strand cDNA was synthesized and second strand cDNA synthesis was subsequently performed. Remaining overhangs were converted into blunt ends. After adenylation of 3′ ends of DNA fragments, adaptor with hairpin loop structure were ligated. Then the PCR was performed. At last, Qubit HS quantification, Agilent 2100 Bioanalyzer/Fragment Analyzer 5300 quality control, the final library size of about 350 bp.

Library construction and high-throughput sequencing on the Illumina Novaseq 6000 platform. Raw reads (Additional file 1: Table S1) of fastq format were firstly processed through primary quality control. The base and reads qualities were assessed by FastQC-0.11.8 (http://www.bioinformatics.babraham.ac.uk/projects/fastqc/). Low quality and adaptor sequences in the raw data were eliminated using Trimmomatic-0.36 (http://www.usadellab.org/cms/uploads/supplementary/Trimmomatic/). In this step, clean reads were obtained by removing read pairs that contain N more than 3 or the proportion of base with quality value below 5 is more than 20%, in any end, or adapter sequence was founded. All the downstream analyses were based on the clean data with high quality.

### SNP calling

DNA were extracted from endosperm of 138A, 398A, 723A, 6A were sampled from 12 days after pollination (DAP) for re-sequencing. DNA degradation and contamination was monitored on 1% agarose gels. DNA purity was checked using the NanoPhotometer® spectrophotometer (IMPLEN, CA, USA). DNA concentration was measured using Qubit® DNA Assay Kit in Qubit® 2.0 Flurometer (Life Technologies, CA, USA). A total amount of 1.5μg DNA per sample was used as input material for the DNA sample preparations. Sequencing libraries were generated using Truseq Nano DNA HT Sample preparation Kit (Illumina USA) following manufacturer’s recommendations and index codes were added to attribute sequences to each sample. These libraries constructed above were sequenced by Illumina NovaSeq platform and 150bp paired-end reads were generated with insert size around 350bp.

The sunflower reference genome (HanXRQr2.0-SUNRISE) was downloaded from NCBI (https://www.ncbi.nlm.nih.gov/assembly/GCF_002127325.2/). The re-sequencing reads were aligned using BWA with default parameters [49]. The SNP calling were performed using Samtools and BCFtools [50]. Only unique mapped reads were remained for SNP calling. SNPs were called per locus. The 12.18–12.95% multi-matched reads were excluded during gene matching analysis. Only the SNPs with the read depth greater than 3 were remained.

### Identification of imprinted genes

Endosperm of SY1, YS1, SY2 and YS2 were sampled at 12 DAP for RNA-seq. The RNA-seq reads were mapped using the software HISAT2 with default parameters [51]. The sunflower reference genome (HanXRQr2.0-SUNRISE) was downloaded from NCBI (https://www.ncbi.nlm.nih.gov/assembly/GCF_002127325.2/). To avoid mapping bias, the SNP sites were converted to 138A, 398A, 723A and 6A nucleotides to constructed four simulated 138A, 398A, 723A and 6A genome. SY1 and YS1 RNA-seq reads were mapped to the simulated 138A and 398A genome. SY2 and YS2 RNA-seq reads were mapped to the simulated 723A and 6A genome. The BAM files generated were transferred to pileup format using Samtools [47]. Then, according to the information of SNPs, we can divide the short sequences aligned at the SNP site from maternal or paternal allele. A series of Perl programs were used to calculate read counts from maternal or paternal allele at each SNPs (Additional file 13). For a gene, the number of reads that mapped to each allele was summed across all SNPs. Only transcripts that had at least 10 reads that could be assigned to a particular allele in each direction of the reciprocal cross could be analyzed. Then, the imprinted genes were identified with the same strategy that was used in previous works [7]. In endosperm, the maternal to paternal allele ratio is 2 to 1. Based on it, two-tailed χ2 test was conducted at each SNP site to test parental bias. *P*-values were converted to Q-values. Genes with parental allele bias (*q* < 0.05) in reciprocal hybrids were classified as low-stringency imprinted genes. To identify the high-stringency imprinted genes, we used more stringent standards. The favorable alleles were at least five times more than those of non-favorable alleles in both directions of a reciprocal cross. For MEGs, the proportion of maternal alleles should be greater than 10/11, i.e. 2 M*5/(2 M*5 + 1P). For PEGs, the proportion of paternal alleles should be greater than 5/7, i.e.5*1P/(2 M + 5*1P).

### GO term and functional category enrichment analysis

GO annotation was performed by InterProScan. The GO term enrichment analysis was conducted for genes included in each cluster using the WEGO 2.0 software (https://wego.genomics.cn/). Imprinted genes and all genes were divided into two groups. GO categories among molecular function and biological process that show significant (*P* < 0.05) enrichment were displayed.

### Identification conserved imprinted genes in sunflower

Potential homologs of Arabidopsis, castor bean, *C. rubella*, rice, maize and sorghum imprinted genes in sunflower were identified using the blastp algorithm. The top five hits were reserved with e-value<1e-5.

### Methylation

DNA from endosperm (SY1 and YS1) and embryo (398A) at 12 DAP were extracted for MethylC-seq. First, the quality and concentration of DNA samples were tested: (1) DNA Nanodrop detection of DNA concentration and purity (OD 260/280 ratio); (2) Agarose gel electrophoresis was used to analyze the degree of DNA degradation and whether there was RNA or protein contamination; (3) Qubit accurately quantified the DNA concentration. DNA samples with OD values between 1.8 and 2.0 and content above 1.0 μg could be used to build the database. After the DNA sample was qualified, a certain proportion of negative control (Lambda DNA) was added. The EZ DNA methylation-Gold Kit (ZYMD RESEARCH) was used for the Bisulfite process. The Bisulfite processed products were amplified and purified by PCR to obtain the final library.

MethylC-seq reads were mapped to the reference using Bismark with --bowtie2 -N 1 -p 4 --phred33-quals [52]. The bulk methylation of embryo and endosperm are calculated by the ratio of the Cs with all the Cs and Ts from all sites with at least 5 reads. A sliding-window approach with window size of 200 bp and slide step of 20 bp to find DMRs were adopted. Then, in each window, the significance bias of methylation level between embryo and endosperm was weighed by *P*-value (Fisher’s exact test). *P*-values were converted to Q-values. Finally, the windows were filtered by the following criteria: FDR < 0.01; the level of methylation between embryo and endosperm differed > 30%. Windows within 200 bp were merged as one DMR.

## Supplementary Information

**Additional file 1: Table S1.** The summary of sequencing data.

**Additional file 2: Table S2.** The summary of imprinted genes identified in 12 DAP sunflower endosperm.

**Additional file 3: Table S3.** The clusters of imprinted genes in 12 DAP sunflower endosperm.

**Additional file 4: Table S4.** The summary of imprinting conservation between sunflower imprinted genes and those in other species.

**Additional file 5: Table S5.** The summary of DMRs between embryo and endosperm.

**Additional file 6: Table S6.** The summary of imprinted genes overlapped with DMRs.

**Additional file 7: Fig. S1.** The expression ratio between the 398A and 138A alleles at all SNP site in in 398A and 138A endosperm.

**Additional file 8: Fig. S2.** The GO annotation of imprinted genes identified in sunflower endosperm.

**Additional file 9: Fig. S3.** The density plot of distance (bp) between imprinted transcripts.

**Additional file 10: Fig. S4.** The expression profile of imprinted genes among tissues.

**Additional file 11: Fig. S5.** The expression levels of imprinted genes in hybrid endosperm.

**Additional file 12: Fig. S6.** The bulk DNA methylation levels in embryo and endosperm.

**Additional file 13.** The Perl scripts for identification of imprinted genes.

## Data Availability

Sequencing datasets produced or investigated in this study are freely available at NCBI (PRJNA740059).
