# Supplementary figures and images for: Epigenetic modifications potentially controlling the allelic expression of imprinted genes in sunflower endosperm
Source: BMC Plant Biol. 2021 Dec 4;21:570. doi: 10.1186/s12870-021-03344-4 (PMC8642925; doi:10.1186/s12870-021-03344-4)

**Table S1. The summary of sequencing data.**


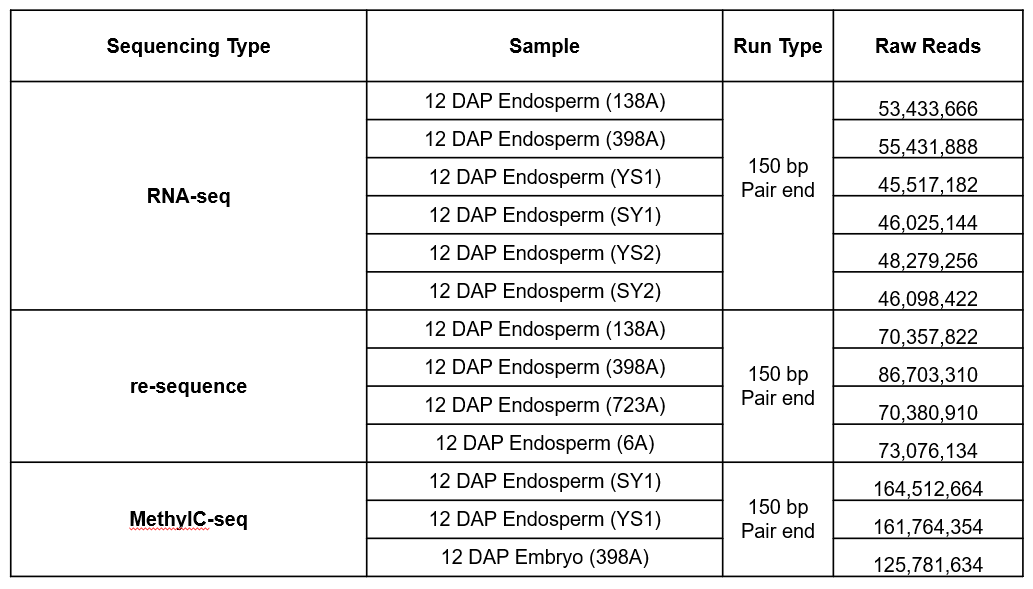

Supplement: Supplementary file 1 — Additional file 1: Table S1. The summary of sequencing data. [file 12870_2021_3344_MOESM1_ESM.docx]

**Table S6: The summary of imprinted genes overlapped with DMRs.**

**
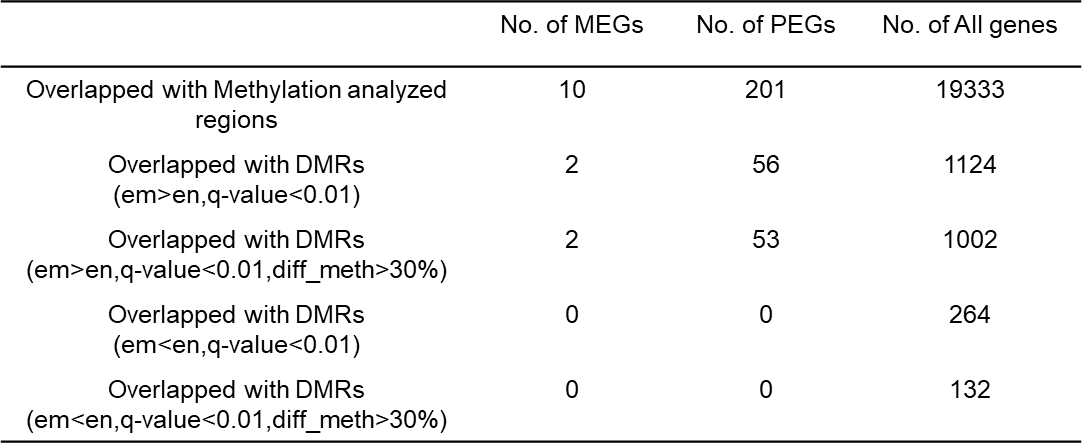
**

Supplement: Supplementary file 6 — Additional file 6: Table S6. The summary of imprinted genes overlapped with DMRs. [file 12870_2021_3344_MOESM6_ESM.docx]
