# Supplementary material for: Epigenetic modifications potentially controlling the allelic expression of imprinted genes in sunflower endosperm
Source: BMC Plant Biol. 2021 Dec 4;21:570. doi: 10.1186/s12870-021-03344-4 (PMC8642925; doi:10.1186/s12870-021-03344-4)
Supplement: Supplementary file 4 — Additional file 4: Table S4. The summary of imprinting conservation between sunflower imprinted genes and those in other species. [file 12870_2021_3344_MOESM4_ESM.docx]

**Table S4: The summary of imprinting conservation between sunflower imprinted genes and those in other species.**

**
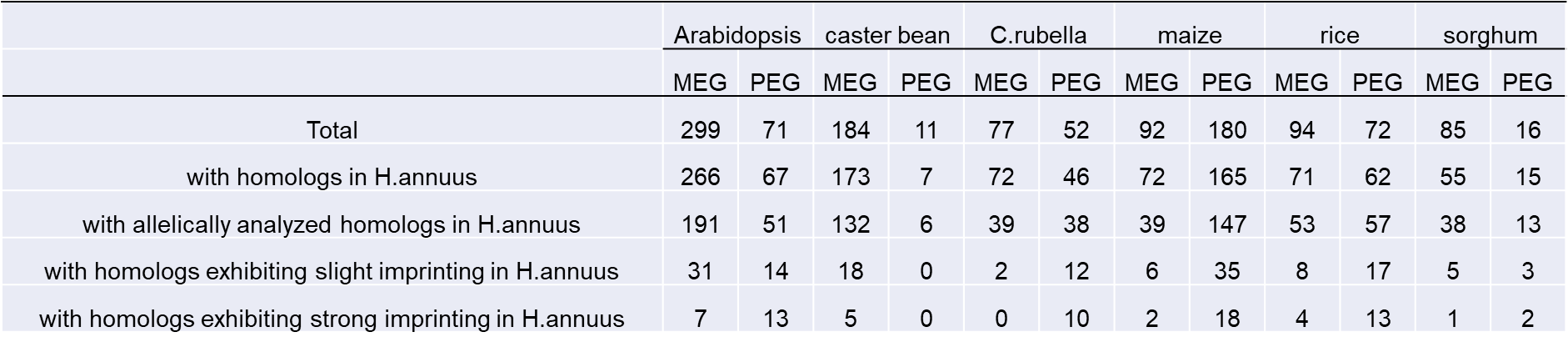
**
