# Supplementary material for: Epigenetic modifications potentially controlling the allelic expression of imprinted genes in sunflower endosperm
Source: BMC Plant Biol. 2021 Dec 4;21:570. doi: 10.1186/s12870-021-03344-4 (PMC8642925; doi:10.1186/s12870-021-03344-4)
Supplement: Supplementary file 7 — Additional file 7: Fig. S1. The expression ratio between the 398A and 138A alleles at all SNP site in in 398A and 138A endosperm. [file 12870_2021_3344_MOESM7_ESM.docx]

**Fig. S1. The expression ratio between the 398A and 138A alleles at all SNP site in 398A and 138A endosperm.**

The reads from the 398A or 138A alleles at all SNP site were summed in RNA-seq data from 398A and 138A endosperm. The blue and orange color represents the proportion of 398A and 138A allele, respectively.
