# Supplementary material for: Epigenetic modifications potentially controlling the allelic expression of imprinted genes in sunflower endosperm
Source: BMC Plant Biol. 2021 Dec 4;21:570. doi: 10.1186/s12870-021-03344-4 (PMC8642925; doi:10.1186/s12870-021-03344-4)
Supplement: Supplementary file 8 — Additional file 8: Fig. S2. The GO annotation of imprinted genes identified in sunflower endosperm. [file 12870_2021_3344_MOESM8_ESM.docx]

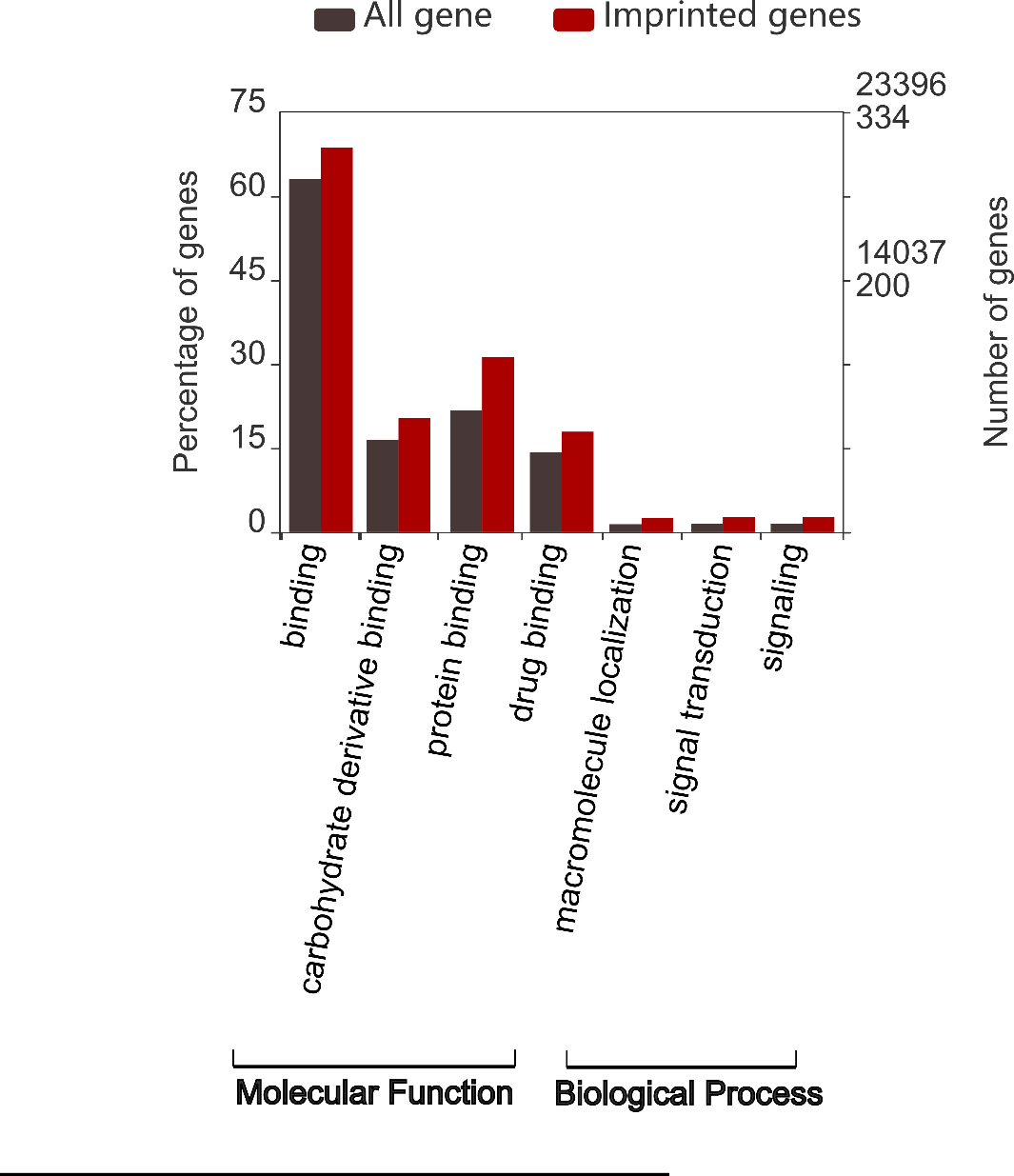


**Fig. S2. The GO annotation of imprinted genes identified in sunflower endosperm.**

The GO term enrichment analysis was conducted for genes included in each cluster using the WEGO 2.0 software (<https://wego.genomics.cn/>). Binding (p-value=0.015); Carbohydrate derivative binding (p-value=0.028); Protein binding (p-value<0.001); drug binding (p-value=0.023); macromolecule localization (p-value=0.046); signal transduction (p-value=0.042); signaling(p-value=0.042).
