# Supplementary material for: Epigenetic modifications potentially controlling the allelic expression of imprinted genes in sunflower endosperm
Source: BMC Plant Biol. 2021 Dec 4;21:570. doi: 10.1186/s12870-021-03344-4 (PMC8642925; doi:10.1186/s12870-021-03344-4)
Supplement: Supplementary file 9 — Additional file 9: Fig. S3. The density plot of distance (bp) between imprinted transcripts. [file 12870_2021_3344_MOESM9_ESM.docx]

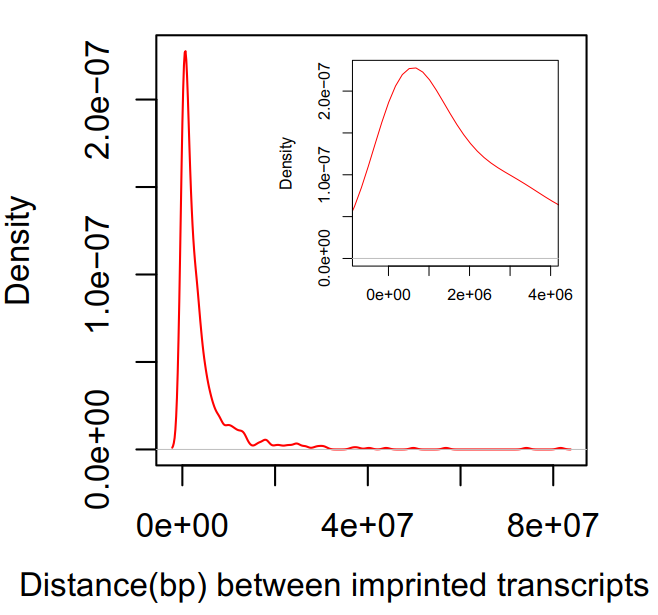


**Fig. S3. The density plot of distance (bp) between imprinted transcripts.**

The distance between each two imprinted genes were calculated. The x-axis represents the distance between two imprinted genes (bp); The y-axis represent the distribution density. The distance within 4,000,000 bp were enlarged and showed in inset plot.
