# Supplementary material for: Epigenetic modifications potentially controlling the allelic expression of imprinted genes in sunflower endosperm
Source: BMC Plant Biol. 2021 Dec 4;21:570. doi: 10.1186/s12870-021-03344-4 (PMC8642925; doi:10.1186/s12870-021-03344-4)
Supplement: Supplementary file 10 — Additional file 10: Fig. S4. The expression profile of imprinted genes among tissues. [file 12870_2021_3344_MOESM10_ESM.docx]

**
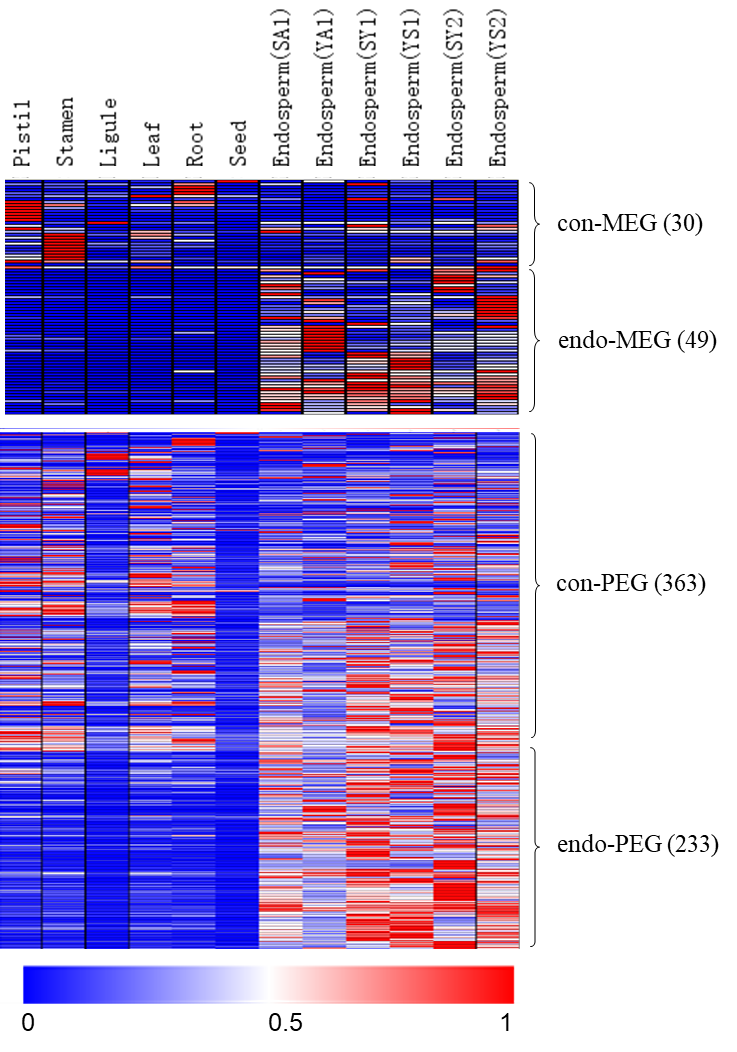
**

**Fig. S4. The expression profile of imprinted genes among tissues.**

Heat map based on the RPKM value normalized by the maximum value of all RPKM values of the imprinted genes over all tissue samples.
