# Supplementary material for: Epigenetic modifications potentially controlling the allelic expression of imprinted genes in sunflower endosperm
Source: BMC Plant Biol. 2021 Dec 4;21:570. doi: 10.1186/s12870-021-03344-4 (PMC8642925; doi:10.1186/s12870-021-03344-4)
Supplement: Supplementary file 11 — Additional file 11: Fig. S5. The expression levels of imprinted genes in hybrid endosperm. [file 12870_2021_3344_MOESM11_ESM.docx]

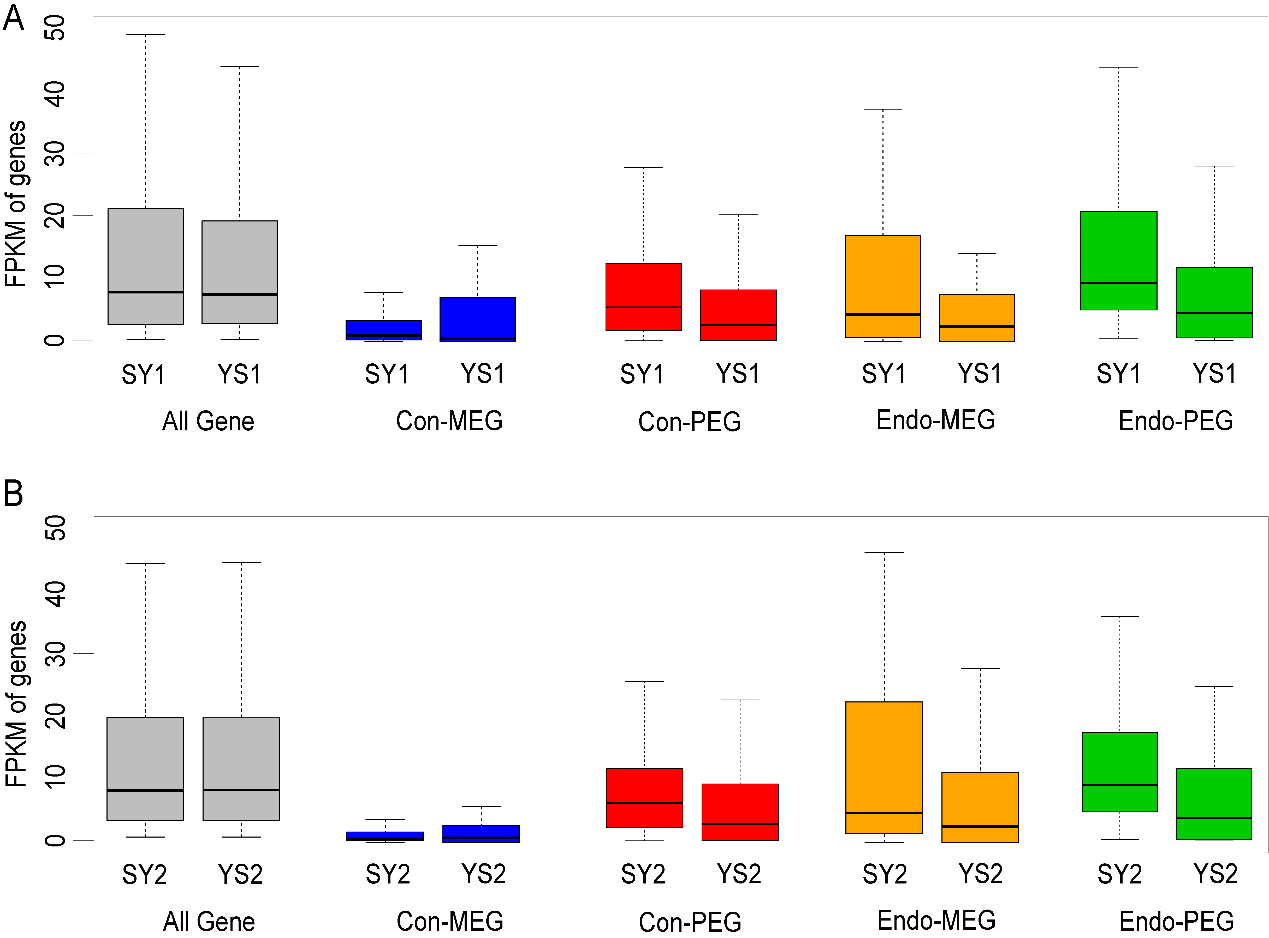


**Fig. S5. The expression levels of imprinted genes in hybrid endosperm.**

The boxplot showed the FPKM value of con-MEG, con-PEG, endo-MEG, endo-PEG and all genes in hybrid endosperm.
