# Supplementary material for: Epigenetic modifications potentially controlling the allelic expression of imprinted genes in sunflower endosperm
Source: BMC Plant Biol. 2021 Dec 4;21:570. doi: 10.1186/s12870-021-03344-4 (PMC8642925; doi:10.1186/s12870-021-03344-4)
Supplement: Supplementary file 12 — Additional file 12: Fig. S6. The bulk DNA methylation levels in embryo and endosperm. [file 12870_2021_3344_MOESM12_ESM.docx]

**Fig. S6. The bulk DNA methylation levels in embryo and endosperm.**

The y axis shows the bulk methylation of 12DAP embryo and endosperm (SY1 and YS1) in CG, CHG and CHH contexts, which are calculated by the ratio of the Cs with all the Cs and Ts from all the analyzable CG, CHG or CHH sites (at least 5 reads per site in all three tissues).
